# Description of a new species of *Calliostoma* (Gastropoda, Calliostomatidae) from Southeastern Brazil

**DOI:** 10.3897/zookeys.224.3684

**Published:** 2012-10-01

**Authors:** Ana Paula S. Dornellas

**Affiliations:** 1 Museu de Zoologia da Universidade de São Paulo, Cx. Postal 42494; 04299-970, São Paulo, SP, Brazil

**Keywords:** Anatomy, *Calliostoma tupinamba*, coastal island, new species, taxonomy

## Abstract

*Calliostoma tupinamba* isa new species from Southeastern Brazil, ranging from southern Rio de Janeiro to northern São Paulo, and found only on coastal islands, on rocks and sessile invertebrates at 3 to 5 meters of depth. Shell and soft part morphology is described here in detail. *Calliostoma tupinamba* is mainly characterized by a depressed trochoid shell; eight slightly convex whorls; a sharply suprasutural carina starting on the third whorl and forming a peripheral rounded keel; and a whitish, funnel-shaped and deep umbilicus, measuring about 5%–10% of maximum shell width. *Calliostoma tupinamba* resembles *Calliostoma bullisi* Clench & Turner, 1960 in shape, but differs from it in being taller and wider, having a smaller umbilicus and lacking a strong and large innermost spiral cord at its base. Finally, an identification key of Brazilian *Calliostoma* species is presented.

## Introduction

The speciose genus *Calliostoma* Swanson, 1840 has a worldwide distribution, occurring from the intertidal zone to depths of several hundred meters (Clench and Turner 1960). The species are frequently found in association with sessile invertebrates, such as hydrozoans, gorgonians and urchins, and many of them are carnivorous (Perron 1975; Perron and Turner 1978; Quinn 1981; Marshall 1988, 1995; Williams et al. 2010; Dornellas and Simone 2011).

An astounding diversity of *Calliostoma* species is found in the Western Atlantic: almost 100 species, 18 of which occur in Brazilian waters (Clench and Turner 1960; Quinn 1992; Rosenberg 2009). *Calliostoma* shells generally have spire typically straight-sided, but may be redounded, and the base ranges from flat to convex, umbilicate or imperforate, sculptured by spiral beaded cords, with a subquadrate aperture and an arched columella. Their ground colors usually are slightly yellowish or brownish, while their secondary coloration consists of white, red or reddish-brown blotches (Hickman and McLean 1990). On anatomical grounds, *Calliostoma* shows some apparently unique features, such as the presence of an ampulla in females, the reduction or lack of cephalic lappets, the presence of a pseudoproboscis and the intestinal loop placed outside of the haemocoel (Randles 1905; Fretter and Graham 1962; Sá and Coelho 1986; Dornellas and Simone in press; Dornellas personal observation).

A new species of *Calliostoma* from Southeastern Brazil is here described, with a detailed morpho-anatomical description. An identification key, based on shell characters of fully grown Brazilian *Calliostoma* species is provided.

## Material and methods

Anatomical abbreviations: **an**, anus; **ap**, ampulla; **aoc**, anterior odontophore cartilage; **bc**, buccal commissure; **bg**, buccal ganglion; **cc**, cerebral commissure; **ccn**, cerebropedal connective; **ce**, cerebral ganglion; **cm**, columellar muscle; **cp**, cerebropleural connective; **ct**, cephalic tentacle; **cv**, ctenidial vein or efferent gill vessel; **dc**, dorsal chamber of buccal mass; **df**, dorsal fold; **el**, esophageal papillae; **es**, esophagus; **eso**, epipodial sense organ; **et**, epipodial tentacle; **ev**, esophageal valve; **ft**, foot; **gi**, gill; **hg**, hypobranchial gland; **Il**, inner lips; **jm**, jaw and peribuccal muscles; **jw**, jaws; **lg**, labial ganglia; **lp**, lateromarginal plate; **m1** to **m11**, odontophore muscles; **mb**, mantle border; **mo**, mouth; **ne**, nerve; **nl**, neck lobe; **od**, odontophore; **om**, ommatophore; **op**, operculum; **opd**, opercular pad; **os**, osphradium; **pb**, pseudoproboscis; **pc**, pericardium; **pg**, anterior furrow of pedal glands; **poc**, posterior odontophore cartilage; **ps**, papillary sac; **ra**, radula; **rc**, radichian; **re**, rectum; **rk**, right kidney; **rn**, radular nucleus; **rt**, radular tissue; **sc**, subradular cartilage; **sg**, skeleton of gill; **sn**, snout; **vc**, ventral chamber of buccal mass; **vf**, ventral fold.

Specimens preserved in 70% alcohol were extracted from their shells and dissected under a stereomicroscope. All drawings were made with a *camera lucida*. For detailed examination of radulae, jaws and protoconch, samples were mounted on stubs, coated with a gold-palladium and observed under a scanning electron microscope. Dimensions for the holotype are given as height X width. Protoconch and shell whorls are counted following Diver (1939) methodology. The list of examined material, housed at MZSP, MNRJ and MOFURG, follows the species description.

**Material** **examined.** *Calliostoma tupinamba* types. Brazil, São Paulo state, Ilhabela island, 1 specimen, MZSP 59090 (Simone, col v/1995); Búzios Island, Aquário coast, MZSP 105574, 07 specimens (Dornellas col., 16/v/2012). *Calliostoma bullisi*. Brazil, Amapá, Cabo Orange holotype USNM 612702. *Calliostoma hassler*. Brazil, Bahia; Belmonte, MZSP 37264, 03 specimens (A. Bodart, col. vii/2003); Alcobaça, MZSP 34885, 11 specimens (Coltro leg.). Espírito Santo; Guarapari, 05 specimens (vi/2006); MZSP 69424, 09 specimens (A. Bodart, col. v/2004); MZSP 57485, 09 specimens (Coltro leg. iv/1992). *Calliostoma depictum*. Brazil, Bahia, Salvador, MZSP 66727, (Linhares, col. vii/1998) 01 specimen. *Calliostoma militare*. Brazil, Rio de Janeiro, off Saquarema, MZSP 66961, 02 specimens (P. Gonçalves col. viii/2002). *Calliostoma brunneopictum*. Brazil, Rio de Janeiro, off Santana, MZSP 73915, 14 specimens (col. xi/1999). *Calliostoma viscardii*. Brazil, Rio de Janeiro, off Saquarema, MZSP 68597, 02 specimens (P. Gonçalves col. vii/2006). *Calliostoma carcellesi*. Brazil, Rio de Janeiro, Arraial do Cabo, MZSP 70459, 01 specimen (col. v/2001). *Calliostoma adspersum*. Brazil, São Paulo, São Sebastião, MZSP 94332, 02 specimens (J. Vaz leg. 03/iii/1998). *Calliostoma rota*. Brazil, São Paulo, Ubatuba, MZSP 38524, 02 specimens (BIOTA-FAPESP col. 10/vi/2001). *Calliostoma jucundum*. Brazil, Rio de Janeiro, Niterói, MZSP 66598, 02 specimens (col. xii/2001). *Calliostoma gemmosum*. Brazil, Bahia, Salvador, MZSP 34850, 02 specimens.

Institutional abbreviations: MNRJ, Museu Nacional da Universidade Federal do Rio de Janeiro, Brazil; MOFURG, Museu Oceanográfico "Prof. E.C. Rios", da Universidade Federal do Rio Grande, Brazil; MZSP, Museu de Zoologia da Universidade de São Paulo, São Paulo, Brazil; NMNH, National Museum of Natural History, Smithsonian Institution, Washington, D.C., USA.

## Systematics

### Clade Vetigastropoda Salvini-Plawen, 1980. Superfamily Trochoidea Rafinesque, 1815. Family Calliostomatidae Thiele, 1924. Genus *Calliostoma*Swainson, 1840

**Type species:** *Trochus conulus* Linné, 1758: 3579 (SD by Herrmannsen 1846: 154).

#### Calliostoma tupinamba sp. n.

urn:lsid:zoobank.org:act:3AD5A1CD-5634-4E17-995F-77784551943F

http://species-id.net/wiki/Calliostoma_tupinamba

Figures 1–9 16–28

Calliostoma ilhabelensis Prado, 2003. (*nomen nudum*)
Calliostoma ilhabelense : Rosenberg 2009. (*nomen nudum*)
Calliostoma jujubinum : Sá and Coelho 1986: 263–271; Rios 2009: 49 (fig. 105) (in part; *non* Gmelin 1791).

##### Type material.

Holotype MZSP 105740 (37.3 mm × 38.2 mm). Paratypes: MZSP 91745, 4 specimens, from type locality. São Paulo state; São Sebastião, Therezinha wreck, 23°54'26"S, 45°27'57"W, 10 m (col. 20.i.2012), MZSP 103766, 2 specimens; Alcatrazes Archipelago, Farol Island, 24°05'44.7"S, 45°42'08.0"W, 6–9 m (coll. 05.x.2011), MZSP 102223, 7 specimens, MNRJ 30603, 1 specimen, MOFURG 51661, 1 specimen; Ilhabela, Saco do Eustáquio, 23°50'11"S, 45°14'21"W, 12 m (col. 09.i.2012), MZSP 105118, 1 specimen; Vitória Island, Saco do Paiá, 23°44.658'S, 45°01.343'W, 6–9 m (15.v.2012), MZSP 105660, 4 specimens, MNRJ 30602, 1 specimen, MOFURG 51662, 1 specimen.

##### Type locality.

Brazil, Rio de Janeiro state, Angra dos Reis, Jorge Grego Island, 23°13'S, 44°08'W, 5–7 m (col. 08–09.viii.2009).

##### Etymology.

Reference to the Ecological Station (Esec) Tupinambás for expeditions along the southeastern coast of Brazil and is an arbitrary combination of letters.

##### Diagnosis.

Shell reaching 38 mm in height, with sharply suprasutural carina starting on the third whorl and forming a large peripheral rounded keel. Umbilicus deep, white, funnel-shaped, narrow (5% to 10% of maximum shell width). Base convex.

##### Description.

**Figures 1–9.**
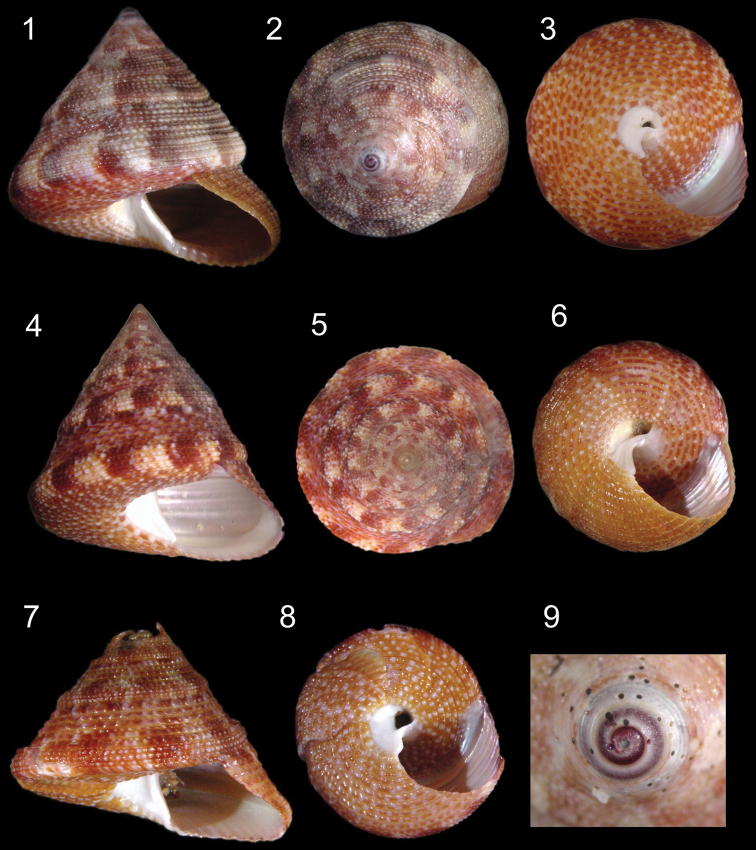
*Calliostoma tupinamba* sp. n. shells. **1–3, 9** Holotype MZSP 105740 **1–3** Apertural, apical and umbilical views, 37.3 mm height **4–6** Paratype MZSP 102223, apertural, apical and umbilical views, 32 mm height **7–8** Paratype MZSP 91745, apertural and umbilical views, 22 mm height **9** Apical view, detail of protoconch.

Shell (Figs 1–9, 16). Attaining 37.6 mm × 38.2 mm; depressed trochoid; 8 whorls, slightly convex. Sharp suprasutural carina starting on third whorl and forming peripheral rounded keel. Basic color of shell pale or tawny brown to pinkish-brown with white and dark red to purple spots and axial flammules, especially on periphery; apex purple; base with numerous white dots on beads. Protoconch (Figs 9, 16) of 0.75 whorls, sculptured with tiny pustules. Transition to teleoconch marked by a weak terminal varix. Teleoconch sculptured by beaded spiral cords (beads crowded and rounded), with one or two smaller intercalary beaded spiral cords between, without a pattern. About 20 beaded cords on the last whorl. Cords on the body whorls becoming remarkably narrower towards periphery. First three whorls of teleoconch with weak axial riblets. First whorl with about 15 axial riblets and two weakly spiral cords that produce reticulate sculpture at their intersections (Fig. 16). The spiral cords become stronger starting on the second whorl. Base convex (flattened in juveniles), with about 15 cords between umbilicus and periphery. Cords equal in size and smoother than that of the teleoconch. Spire angle ~80º. Aperture subquadrate, with base round in adults but flat in juveniles; outer lip at ~55º angle from base. Columella truncated, heavily arched, thickened, white, terminating in a rounded denticle. Umbilicus deep, white, funnel-shaped, 5% to 10% of maximum shell width.

**Figures 10–15.**
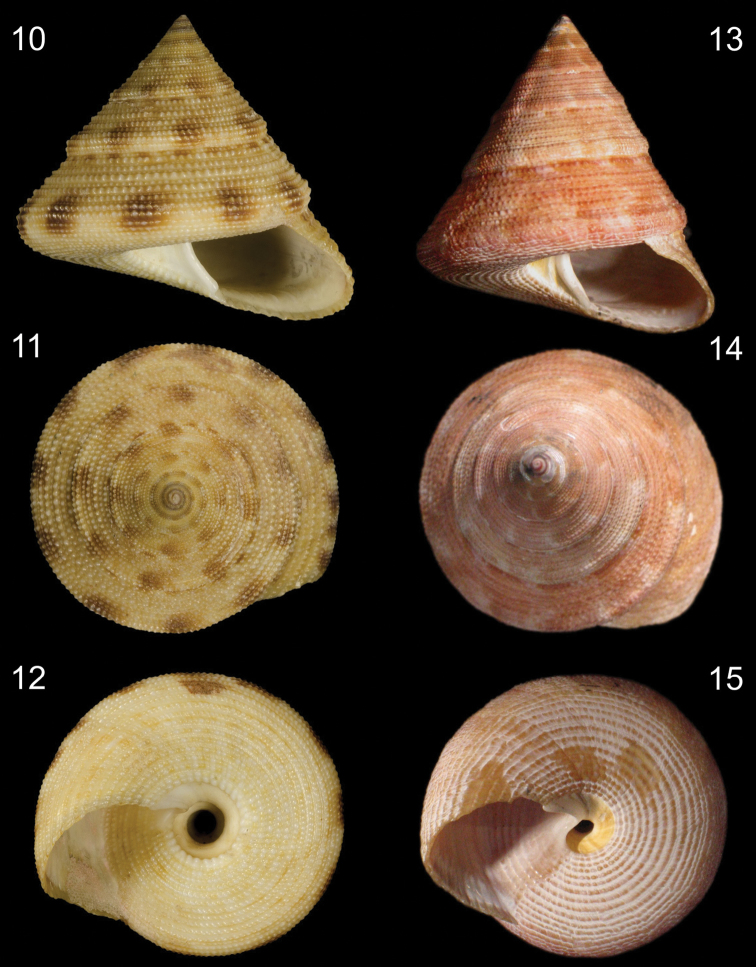
Shells of *Calliostoma bullisi* and *Calliostoma hassler*. **10–12** *Calliostoma bullisi* holotye NMNH 612702, frontal, apical and umbilical views, length = 24.2 mm, courtesy of NMNH **13–15** *Calliostoma hassler*, MZSP 49814, frontal, apical and umbilical views, length =34 mm.

**Figures 16–19.**
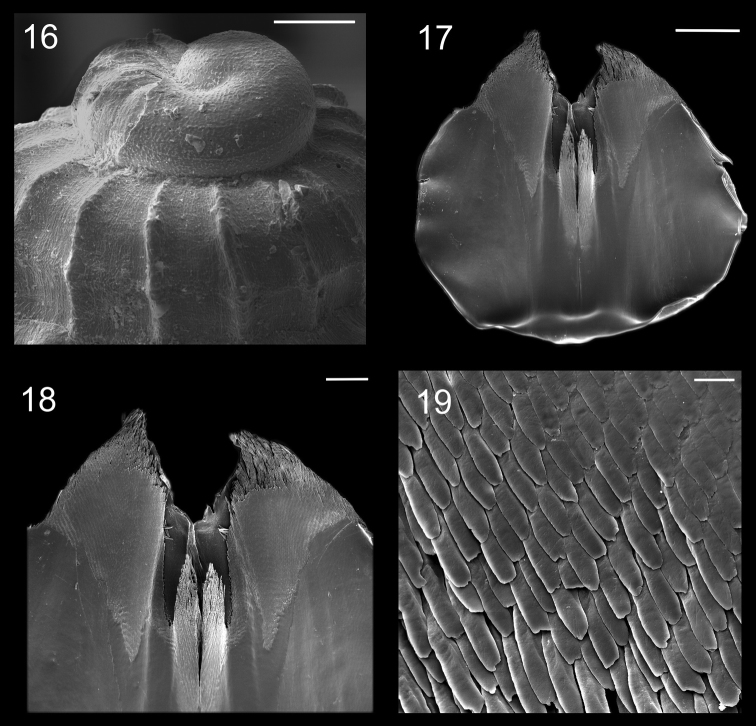
Protoconch and jaws of *Calliostoma tupinamba* holotype in SEM **16** Protoconch profile and apical view, scale bar = 100 µm **17–19**. Jaws **17** Ventral view, scale bar = 500 µm **18** Ventral view of anterior edge, scale bar = 200 µm **19** Detail of anterior area, scale bar = 20 µm.

Head-foot (Figs 29–30, 32). Total length two whorls. Head rounded located approximately at anterior end of head-foot. Snout reddish, wide, cylindrical; distal end slightly wider than base; dorsal surface papillated, with short, rounded and white papillae; distal surface folded. Outer lips with pseudoproboscis (Fig. 32: pb). Mouth circular, located in middle of ventral surface of snout. Cephalic lappets absent. Cephalic tentacles usually twice larger than snout, sometimes asymmetrical, reddish, dorsoventrally flattened, grooved, narrowing gradually up to lightly pointed tip. Ommatophores located at outer base of cephalic tentacles, ~1/3 of tentacles’ length. Eyes dark and round, at tip of ommatophores.

Foot thick, occupying ~3/4 of total head-foot length; reddish colored; dorsal region covered by numerous white papillae. Epipodium (Figs 29–30) surrounding lateral-dorsal region of foot, equidistant from sole and base of ommatophore. Opercular pad (Fig. 29: opd) located in the middle of dorsal region; rounded, edge free on posterior area; posterior end with several V-shaped furrows, apex pointed toward posterior end and pair of longitudinal furrows on median line. Furrow of pedal glands present along entire anterior edge of foot (Fig. 30: pg); single aperture of pedal glands located in median region of furrow. Anterior third of epipodium formed by well-developed neck lobes (Figs 29–30: nl); left neck lobe smooth, right neck lobe with fine digitations. Remaining 2/3 of epipodium relatively low, thick; 4 pairs of slender tentacles inserted at its distal edge on each side and located approximately equidistant, well away from each other, symmetrical on both sides, becoming shorter towards posterior end. Epipodial sense organ present at base of each tentacle, indistinguishable from the foot papillae on the lateral sides of the foot. Pair of columellar muscles thick, ~¼ whorl, fused with each other in median line.

Operculum. About 13 mm in diameter, closing entire aperture. Yellowish gold, thin, corneous, multispiral, nucleus central. Inner side convex, outer side concave.

Mantle organs (Fig. 31). Pallial cavity ~3/4 whorl. Mantle border (mb) thick, white with irregular band of brown; anterior end papillated, occupying ~1/3 of mantle border. Gill located on left side of pallial cavity, occupying almost its entire length, projecting anteriorly and sustained by gill rod (sg) and suspensory membrane. Anterior end of gill narrow, with pointed tip, gradually increasing towards narrow posterior end. Osphradium rounded located at base of gill road. Afferent gill vessel ~2/3 of gill’s length, arising from transverse pallial vessel, running in distal region of central axis of gill. Transverse pallial vessel arising off from left nephrostome, discharging in afferent gill vessel. Ctenidial vein (cv) (= efferent gill) vessel about 1/3 longer than afferent vessel, running in basal region of gill central axis; its posterior 1/9 free from gill filaments, lying parallel, at left from afferent vessel up to pericardium. Hypobranchial gland (hg) on both sides of rectum; more developed on left side. Rectum ~¼ of pallial cavity width, sigmoid on posterior region under kidneys, gradually straightening towards the anus. Anus siphoned, smaller than rectum’s width; pleated, short free end, located on anterior right side of pallial cavity. Kidneys posteriorly located in pallial cavity, ~1/3 of rectum’s length.

Visceral mass. Not studied.

Circulatory and excretory systems (Fig. 31). Pericardium (pc) located between pallial cavity and visceral mass, immediately posterior to kidneys, close to median line; its left side receiving ctenidial vein and right side receiving right pallial vein.

Papillary sac (ps) of left kidney ~1/3 of rectum’s length; oval, with wide base; gradually narrowing towards anterior, ending at left of nephrostome; inner wall with numerous thin and long papillae projecting inward from all surfaces. Right kidney (rk) divided in two regions: anterior region hollow, a tube ~1/3 of rectum’s length and ~1/2 width of papillary sac in male; in female called ampulla (ap), large, oval, hollow, filled by mucus, ~3/4 larger than papillary sac; kidney expanding ventrally, covering right half of surface of adjacent visceral hump; posterior region spreading around visceral mass immediately beneath mantle, encircling inner surface of columellar muscle.

**Figures 20–28.**
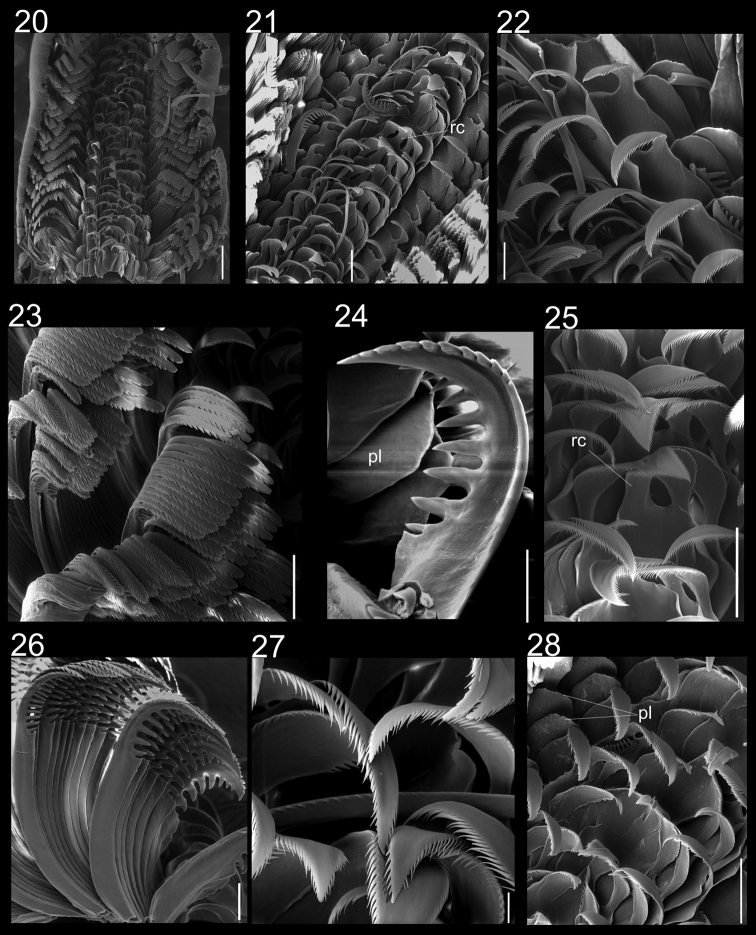
Radulae of *Calliostoma tupinamba* holotype and paratype MZSP 102223 in SEM. **20** Ventral view of radular ribbon, scale bar = 200 µm **21** Ventral view of middle region, scale bar = 100 µm **22** Detail of outermost lateral tooth and lateromarginal plate, scale bar = 50 µm **23** Marginal teeth, ventral view, scale bar = 100 µm **24** Detail of innermost marginal tooth, scale bar = 50 µm **25** Rachidian and lateral teeth, scale = 100 µm **26** Marginal teeth, lateral view, scale bar = 50 µm **27** Detail of lateral teeth, scale = bar 20 µm **28** Rachidian, lateral teeth and lateromarginal plate, scale bar = 100 µm.

Digestive system (Figs 17–28; 32–38). Oral tube ~1/2 of odontophore in length and width; walls with circular muscles. Jaws triangular in shape, dark brown; very long denticles on anterior end, projecting in tufts (Figs 17–19). Pair of dorsal folds starting posteriorly to jaws (Fig. 33: dc), each dorsal fold bending, partially overlapping, forming two slits; upper slit rounded, ventral slit triangular. Series of transverse muscles separating outer surface of esophagus from odontophore. Odontophore about twice longer than snout. Odontophore muscles (Figs 34–38): **m1**, series of small muscles connecting buccal mass with adjacent inner surface of snout and haemocoel; **m1d**, pair of small dorsal retractors muscles, originating in postero-lateral region of mouth sphincter (Figs 35–36: mc), inserting itself in antero-lateral edge of posterior cartilage; **m1v**, pair of small ventral protractors muscles of odontophore, originating on surface central of oral tube, running posteriorly away from each other, inserting in postero-ventral region of odontophore, in median surface of posterior cartilages; **m2a**, anterior retractor muscles of odontophore, originating in antero-lateral surface of anterior cartilages and inserting in hemocelic lateral walls; **m2b**, posterior retractor muscles of odontophore, originating in lateral surface of anterior cartilages, posterior to m2a, inserting in haemocelic lateral walls; **m4**, broad pair of dorsal tensor muscles of radula, subradular membrane, originating partly in anterior cartilages, along their ventral surfaces at some distance from median line, and partly in posterior cartilages, in their posterior, lateral surfaces, surrounding anterior cartilages lateral, ventral surfaces, and inserting along subradular membrane, in its dorsal region exposed inside buccal cavity, with portion in radular ribbon in its region preceding buccal cavity; **m5**, pair of large accessory dorsal tensor muscles of radula, originating in ventral surface of posterior cartilages, running towards median dorsal region, subsequently running anteriorly, inserting in in posterior region of radular ribbon; **m6**, horizontal muscle, uniting both anterior cartilages almost along entire ventral edge, except for short posterior region, in their external surface; **m7a**, very long, thick pair of muscles, originating in hemocelic ventral surface, running dorsally, inserting in radular sac, posteriorly to insertion of m5; **m7b**, pair of muscles originating in lateral inside wall of radular sac, dividing into three thin beam muscles, two of them inserting in posterior cartilage and other entering through m10l; **m8**, pair of broad approximator muscles of cartilages, originating in anterior cartilages, along its lateral surface, posterior to insertion of jm, running posterior, decreasing gradually, inserting in middle region of anterior surface of posterior cartilages; **m10**, pair of broad ventral protractor muscles of odontophore, originating from ventral region of mouth and buccal sphincter, running posteriorly, inserting in anterior region of posterior cartilage just ventral to m8 insertion; **m10l**, pair of broad lateral protractor muscles of odontophore, originating from lateral oral cavity, inserting in outside of anterior region of posterior cartilage; **m11**, two pairs of ventral tensor muscles of radula, originating in middle region of ventral surface of posterior cartilage, one separated from other by distance equivalent to their width, running anteriorly covering m6, anterior cartilage’s ventral surface, becoming wider in anterior region, inserting in subradular membrane distal edge; **m11a**, very long, thin pair of oblique tensor muscles of radula, originating in hemocelic anterior surface near pleural ganglia, running dorsally through between anterior edge of anterior cartilages, inserting in subradular membrane distal edge; **jm**, jaw muscles, originating gradually from dorsal surface of oral tube, close to median line, running divergently towards posterior and sides, inserting themselves in latero-ventral surface of the anterior cartilage; **jma**, dorsal jaw pair muscles, originating in antero-dorsal region of anterior cartilage, running dorsally for short distance, surrounding oral cavity; **ml**, two pairs of wide and thick lateral muscles, originating in hemocelic lateral wall, running straightly forward, internal through the side wall of buccal cavity, inserting themselves in median line of lateral surface of anterior cartilage.

**Figures 29–31.**
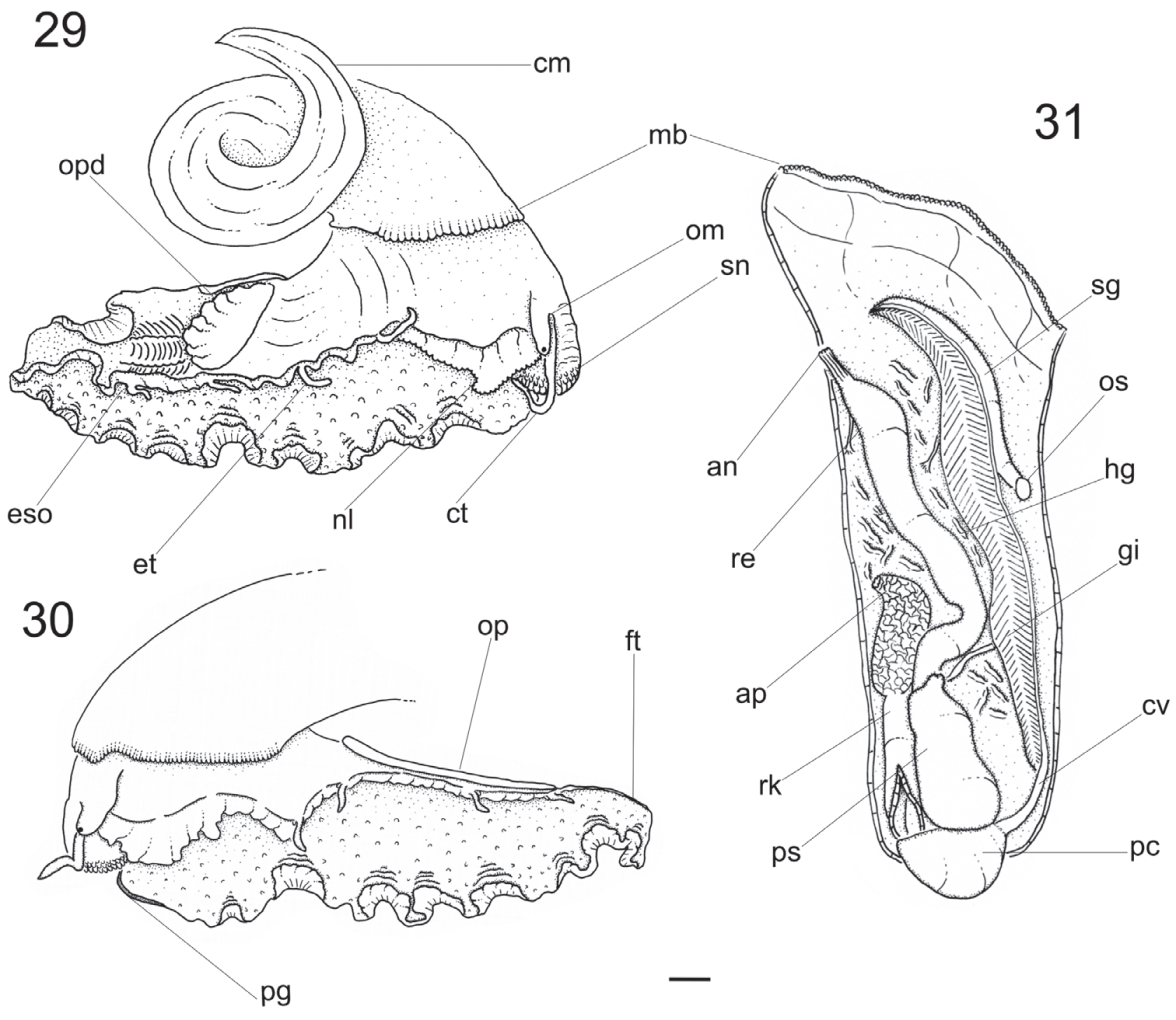
*Calliostoma tupinamba* anatomy, scale bar = 2 mm. **29–30** Head-foot, right and left sides **31** Pallial cavity roof, ventral view.

**Figures 32–36.**
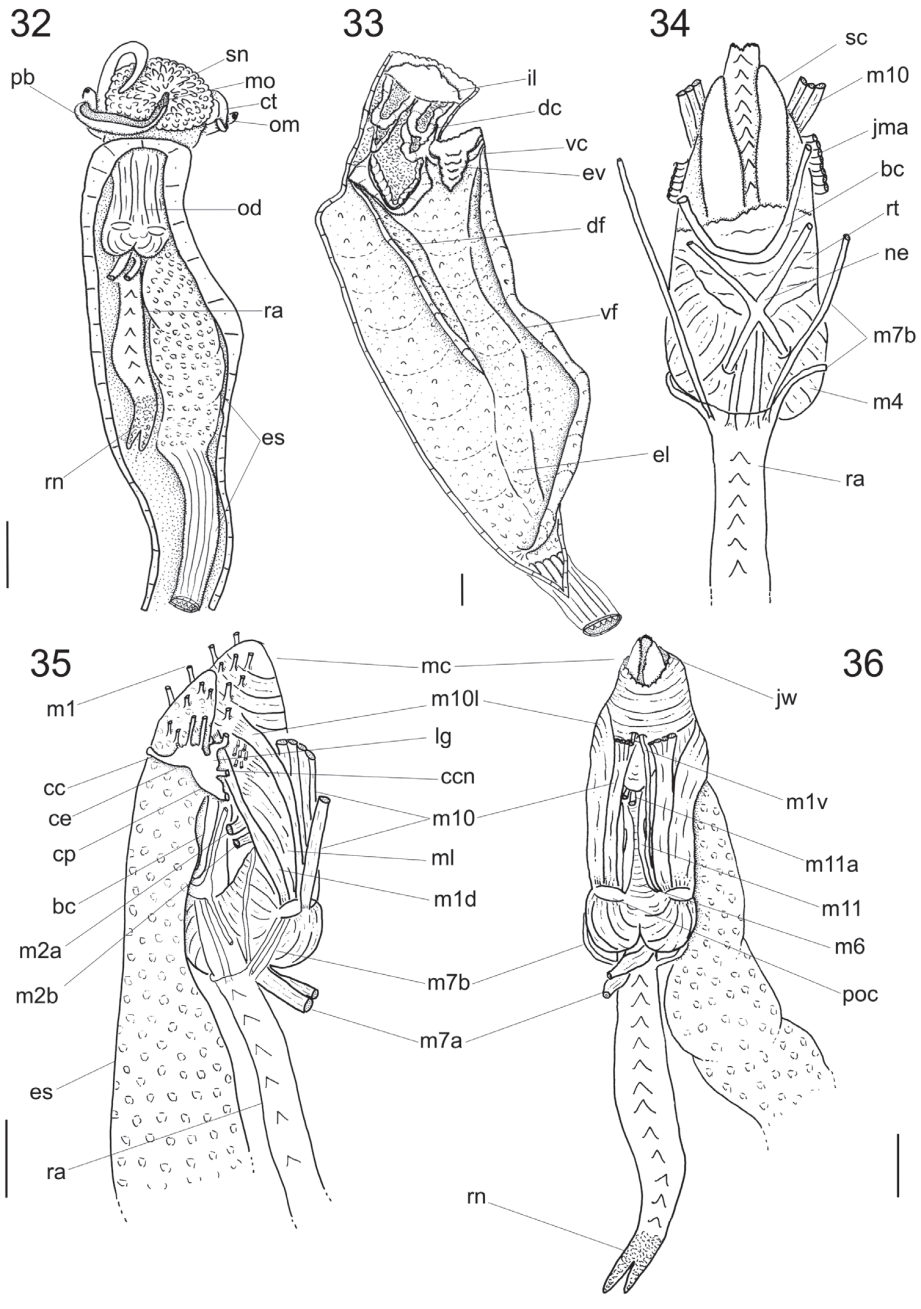
*Calliostoma tupinamba* anatomy. **32** Head and hemocel, ventral view, scale bar = 5 mm **33** Buccal cavity and esophagus opened longitudinally, ventral-inner view, odontophore removed **34** Odontophore, dorsal view **35–36** Buccal mass and central nervous system, right and ventral views. Scales bar = 2 mm.

**Figures 37–38.**
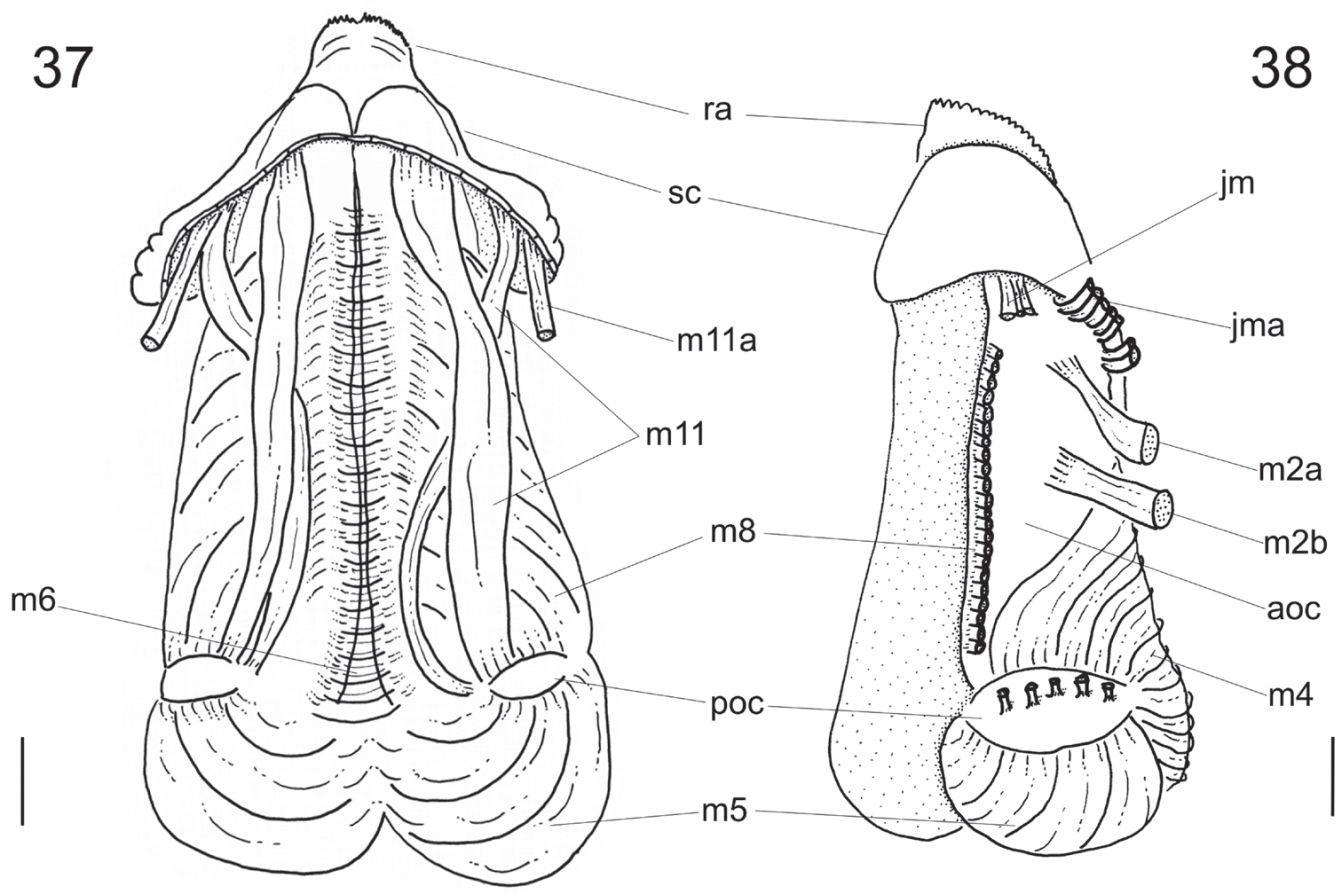
*Calliostoma tupinamba* odontophore, scale bar = 1 mm. **37** Ventral view, subradular cartilage opened longitudinally, m10 and m10l muscles removed **38** Left view, m8, m10 and m10l muscles removed.

Radula (Figs 20–28): symmetrical, arched. **Radichian** (Figs 21, 25: rc) ~1/10 of total radula width, with very large base slightly rounded in shape; slender shaft; cutting edge turned posteriorly, tip narrowly tapered; finely serrated with delicate, thin, pointed and slightly elongated cusps on both sides; apical portion short, with rounded tip. **Lateral teeth** (Figs 21, 22, 27, 28): four pairs, ~1/3 of total radular width; shaped similarly to radichian, but with narrower base; shaft finely denticulate with long, slender cusps, becoming very slender on outer laterals; two pairs of lateromarginal plates (Figs 24, 28: pl) between innermost marginal teeth and outermost lateral teeth. **Marginal teeth** (Figs 23, 24, 26): about 30 teeth, occupying more than ½ of total radula width; more than twice as tall and thinner (~½) than lateral and radichian teeth; innermost pair greatly enlarged (Fig. 24), with broad base and coarsely serrated and hooked cusps; cusps curved, inner ones twice as large as outer ones; succeeding inner marginals of uniform shape; outer marginals becoming slender towards outermost region; food groove on anterior edge of shaft, below the posterior secondary cusps.

Salivary gland indistinguishable from dorsal region of buccal cavity; salivary aperture in mid-dorsal region of buccal cavity, on dorsal folds. Four dorsal fold channels; two of them are a continuation of dorsal folds in mid esophagus (Fig. 33: df); ventral food channel continuing ventral esophageal fold; esophageal valve (Fig. 33: ev) located on ventral food channel; mid esophagus with two esophageal folds, a higher ventral fold and right dorsal fold. Posterior esophagus narrow; inner surface with some longitudinal, narrow folds. Esophagus insertion and stomach not observed. Rectum, anus described above (pallial organs).

Genital system: not observed due to damage during extraction of soft parts.

Central nervous system (Figs 34–35): Nerve ring surrounding anterior half of buccal mass. Cerebral ganglia (Fig. 35: ce) rounded, located on lateral region of buccal mass and occupying ~1/3 of it; commissure thick, long; both dorsoventrally flattened cerebropleural and cerebropedal connectives (Fig. 35: cp, ccn) long and thin, originating in anterior region of cerebral ganglia, running ventrally and back to pedal and pleural ganglia. Cerebropleural connective twice as thick as cerebropedal. Labial ganglia (Fig. 35: lg) very small, ~1/5 of cerebral ganglia, located in ventro-lateral region of buccal mass, anteriorly to cerebral ganglia; linked to cerebral ganglia by short cerebrolabial connective. Buccal ganglia about same size as labia ganglia; located posteriorly to cerebral ganglia; connected united to cerebral ganglia by buccolabial connective; buccal commissure (Fig. 34: bc) thick, lying dorsally to radular sac.

##### Distribution.

From southern Rio de Janeiro to northern São Paulo, 23° to 24°S, 44° to 45°W, only on coastal islands (from Alcatrazes Archipelago to Jorge Grego Island).

##### Habitat.

On rocks and sessile invertebrates, at 3–5 meters.

## Discussion

The overall shell shape of *Calliostoma tupinamba* closely resembles *Calliostoma bullisi* Clench & Turner, 1960 from northern Brazil. It differs in being taller and wider: the holotype of *Calliostoma bullisi* (Figs 10–12) is a 2 cm tall, has a convex base and a well-developed and rounded peripheral keel, indicating an adult stage, while all examined specimens of *Calliostoma tupinamba* of 2 cm are juveniles, showing a flattened base and a less developed peripheral keel (Fig. 7). The base of the shell of *Calliostoma bullisi* has a strongly and larger innermost spiral cord (Fig. 12). When it is present in *Calliostoma tupinamba*, this cord is half the size from that of *Calliostoma bullisi*. The umbilicus of *Calliostoma tupinamba* is much narrower, occupying only 5% to 10% of the maximum shell width, while in *Calliostoma bullisi* the umbilicus occupies 20%. Unfortunately, there is no reference in the literature regarding the soft parts’ anatomy of *Calliostoma bullisi*: Clench and Turner (1960) described only the head-foot morphology, but without illustration of it differentiating apparently by having uniform red brown tentacle while *Calliostoma tupinamba* has a thin darker longitudinal line in median dorsal region of tentacle. The radula of the holotype of *Calliostoma bullisi* was illustrated with a drawing by Clench and Turner (1960: 5, pl. 5, fig. 1). The radula of *Calliostoma tupinamba* differs from *Calliostoma bullisi* by having slender rachidian and lateral shafts.

The protoconch sculpture with a honeycomb pattern is diagnosed for calliostomatids (Hickman and McLean 1990). Marshall (1995) also observed this pattern in an excellent revision of calliostomatids from New Caledonia. *Calliostoma tupinamba* has not such a pattern, which is also absent in *Calliostoma torrei* Clench & Aguayo, 1940 (following Harasewych 2006), *Calliostoma depictum* Dall, 1927 and *Calliostoma adspersum* (Philippi, 1851) (Dornellas and Simone in press). As protoconch sculpture descriptions are lacking in major revisions of the fauna of the western Atlantic (see Clench and Turner 1960; Quinn 1992) it is hard to know whether the honeycomb pattern is present in the western Atlantic *Calliostoma*.

*Calliostoma tupinamba* differs from *Calliostoma hassler* Clench & Aguayo, 1939 (Figs 13–15) from Southeastern Brazil by being wider, having spiral cords with coarser beads and a uniform pattern of mottled color. Anatomically, the snout is more papillated on the dorsal surface in *Calliostoma tupinamba*. The rachidian tooth of *Calliostoma tupinamba* has a rounded base, while the tooth of *Calliostoma hassler* is triangular. Moreover, there are differences in the odontophore’s muscles: *Calliostoma hassler* has not a jma pair and a thicker ml pair (Dornellas and Simone in press).

In a revision of the Western Atlantic’s *Calliostoma*, Clench & Turner (1960) allocated some species such as *Calliostoma jujubinum*, *Calliostoma bullisi* in the subgenus *Elmerlinia*. This taxon is characterized by the triangular shape of the jaws, with denticles projecting in tufts on the distal edge, and by the two lateromarginal plates in the radula. The same pattern is observed in *Calliostoma tupinamba* and *Calliostoma hassler* and, therefore, it seems to be closely related with species of *Elmerlinia*.

### Key for the identification of fully grown Brazilian *Calliostoma* species based on shell characters

**Table:** 

1	Concave to rounded whorls	2
–	Convex to flat whorls	3
2(1)	Slightly concave whorls. Umbilicate. Base smooth, with reddish brown, non-interrupted concentric bands (Quinn, 1992: Figs 88–89; Rios, 2009: fig. 109)	*Calliostoma axelsonni* Quinn, 1992
–	Greatly rounded convex whorls. Imperforate, with umbilical chink. Base with spiral cords and with sp (Fig. 39)	*Calliostoma depictum* Dall, 1927
3 (1)	Spire taller than wide	4
–	Spire about as high as wide	10
4 (3)	Spiral cords with conical beds	5
–	Spiral cords with rounded beds	7
5 (4)	Suture canaliculated. Large shells, up to 40 mm height (Fig. 40)	*Calliostoma militare* Ihering, 1907
–	Suture indistinct, non-canaliculated. Small shells, up to 9	6
5 (5)	Columella nearly straight. Shell uniformly colored, without darker patches (Clench and Turner 1960: plate 36)	*Calliostoma echinatum* Dall, 1881
–	Columella curved. Shell ivory colored with irregular patches of brown (Fig. 41)	*Calliostoma brunneopictum* Quinn, 1992
6 (4)	Teleoconch with shouldered whorls. Suprasutural cords smooth and keeled (Rios, 2009: fig. 120)	*Calliostoma nordenskjordi* Strebel, 1908
–	Teleoconch with almost 6 flat sided whorls, not forming shoulders. Suprasutural cords beaded	8
7 (7)	Interspaces of first whorls lacking axial sculpture, subsequent whorls with low folds. Height up to 13 mm (Fig. 42)	*Calliostoma viscardii* Quinn, 1992
–	All interspaces between spiral cords with oblique axial riblets or low plicae. Shell large, up to 27 mm	9
8 (8)	Shell wide, spire angle 55° to 70° (Fig. 43)	*Calliostoma carcellesi* Clench & Aguayo, 1940
–	Shell narrow, spire angle 35° to 40° (Quin, 1992: Figs 29–30)	*Calliostoma moscatelli* Quinn, 1992
9 (3)	All whorls with a distinct shoulder angulation	11
–	Whorls not shouldered, with convex or flat sides	13
10 (10)	Peripheral keels beaded (Fig. 44)	*Calliostoma adspersum* (Philippi, 1851)
–	Peripheral keels non-beaded, weakly undulated to smooth	12
11 (11)	Circumbasal cords absent or very weak; interspaces between peripheral keels nearly straight (Clench and Turner 1960: plate 18)	*Calliostoma coppingeri* (Smith, 1880)
–	Circumbasal cords weak to strong; interspaces between peripheral keels concave (Fig. 45)	*Calliostoma rota* Quinn, 1992
12 (10)	Imperforate	14
–	Umbilicate	15
13 (13)	Whorl periphery with three widely spaced spiral cords (Quinn, 1992: Figs 27–28)	*Calliostoma tenebrosum* Quinn, 1992
–	Whorl periphery with two spiral cords (Fig. 46)	*Calliostoma jucundum* (Gould, 1849)
14 (13)	Three reddish brown bands encircling each whorl; apex of shell deep purple (Fig. 47)	*Calliostoma gemmosum* (Reeve, 1842)
–	Absence of contrasting colored bands encircling each whorl	16
15	Shell narrow, spire angle ~60°. Final portion of columella arched (Figs 13–15)	*Calliostoma hassler* Clench & Aguayo, 1939
–	Shell wide; spire angle 70° to 80°. Final portion of columella truncated	17
16	Innermost spiral cord at base strong and large. Umbilicus broad (Figs 10–12)	*Calliostoma bullisi* Clench & Turner, 1960
–	Innermost spiral cord at base very weak or absent. Umbilicus narrow (Figs 1–9)	*Calliostoma tupinamba* Dornellas sp. n.

**Figures 39–47.**
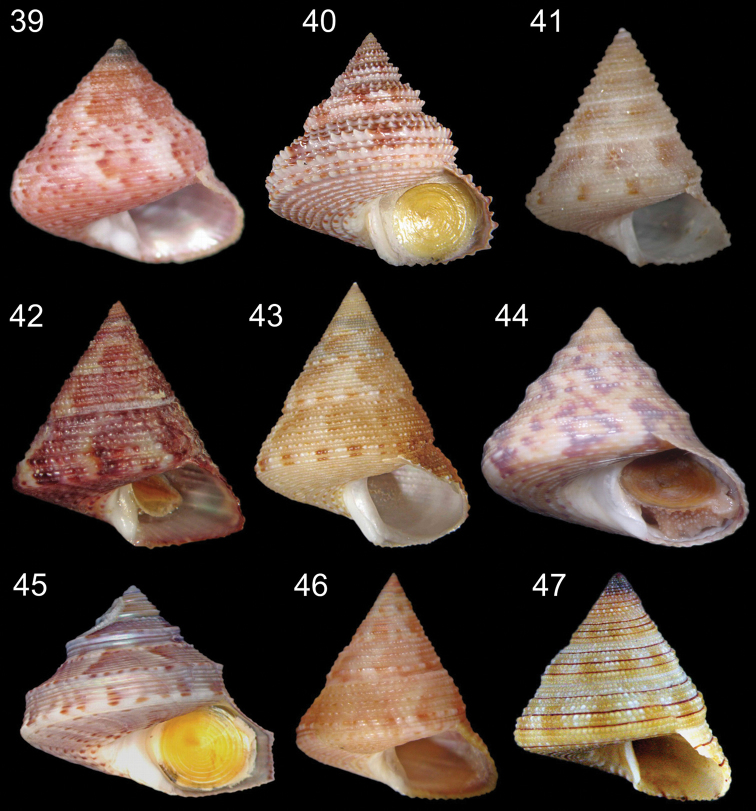
*Calliostoma species* in frontal view. **39** *Calliostoma depictum* MZSP 66727, length = 13.3 mm **40** *Calliostoma militare* MZSP 66961, length = 31.8 mm **41** *Calliostoma brunneopictum* MZSP 73915, length = 9.1 mm **42** *Calliostoma viscardii* MZSP 68597, length = 11.3 mm **43** *Calliostoma carcellesi* MZSP 70459, length = 22.2 mm **44** *Calliostoma adspersum* MZSP 94332, length = 16.5 mm **45** *Calliostoma rota* MZSP 38524, length = 8.9 mm **46** *Calliostoma jucundum* MZSP 66598, length = 26 mm **47** *Calliostoma gemmosum* MZSP 34850, length = 12.6 mm.

## Supplementary Material

XML Treatment for Calliostoma tupinamba
